# Impact of the COVID-19 Pandemic on the Mental Health of College Students: A Systematic Review and Meta-Analysis

**DOI:** 10.3389/fpsyg.2021.669119

**Published:** 2021-07-14

**Authors:** Yang Li, Aiwen Wang, Yalin Wu, Nana Han, Huiming Huang

**Affiliations:** ^1^Faculty of Sports Science, Ningbo University, Ningbo, China; ^2^Research Academy of Grand Health, Ningbo University, Ningbo, China; ^3^Henan Technician College of Medicine and Health, Kaifeng, China

**Keywords:** COVID-19, mental health, college students, depression, anxiety, meta-analysis

## Abstract

**Background:**

The coronavirus disease (COVID-19) pandemic has been spreading and brought unprecedented psychological pressure on people across the entire globe since December 2019.

**Objectives:**

To synthesize the existing evidence of the prevalence of mental health status during the epidemic and provide the basis for mental health education.

**Materials and methods:**

The literature search was conducted in nine databases from December 2019 to October 2020. The risk of bias for each study was assessed, and the random-effects meta-analysis was used to estimate the prevalence of specific mental health problems. The review protocol was registered in PROSPERO with the registration number CRD42020208619.

**Results:**

About 27 studies were included in the analysis with a total of 706,415 participants combined, and 14 mental health problems were gathered. Meta-analysis showed that the prevalence of depression was 39% (95% CI: 27–51%) and that of anxiety was 36% (95% CI: 26–46%). Subgroup analysis indicated that the prevalence of depression and anxiety varied among nations and due to the survey date. The prevalence of depression (60%, 95% CI: 46–74%) and anxiety (60%, 95% CI: 46–74%) in non-Chinese college students was higher than those in Chinese college students (26%, 95% CI: 21–30% and 20%, 95% CI: 14–26%). The proportion of depression (54%, 95% CI: 40–67%) and anxiety (37%, 95% CI: 26–48%) was higher after March 1 than before it (21%, 95% CI: 16–25% and 19%, 95% CI: 13–25%).

**Conclusions:**

The meta-analysis results presented that the prevalence of depression (39%) or anxiety (36%) among college students greatly increased during the COVID-19 pandemic. In addition, the mental health of college students is affected by the nations and the survey date. It was necessary to take measures to reduce mental health risks during the pandemic.

## Introduction

The coronavirus disease (COVID-19) rapidly spread to other areas in China and other countries (Hajivalili et al., 2020; World Health Organization, 2020) since its outbreak in Wuhan, China. The COVID-19 has triggered a global health crisis and is a major public health emergency of international concern (PHEIC) all over the world, which not only threatens the lives of people but also affects their mental health (World Health Organization, 2005, 2020; Zhong et al., 2021). During the pandemic, some people have experienced relatively higher emotional irregularities (e.g., panic, excessive anxiety, irritability, and other psychological reactions) while some people suffered from cognitive imbalances; as a result, their attention and memory may be influenced by repeated stimulation of a large amount of information. Some of them may have changed their behaviors considerably while some have expressed somatic reactions, such as insomnia, stomach pain, and diarrhea (Amerio et al., 2020; Chan and Kuan, 2020; Ren et al., 2020; Yedemie, 2020; Zhao et al., 2020; Zhong et al., 2021). Apropos physiological and psychological responses are normal reactions in dealing with public health emergencies, which are conducive to adapting to the environment. However, overreactions can increase the psychological burden and be hazardous to physical and mental health (Rosenbaum, 2010; Fergusson et al., 2014).

A study, including 992 respondents in China, found that 69% of the respondents were in the high-risk or medium-risk in seven psychological dimensions (mental status, knowledge of stress management, behavioral patterns, risk perception, academic stress, family relationships, and peer relationships) (Chen B. et al., 2020). A mental health survey, including 505 Bangladeshi college students during COVID-19, showed that 28.5% of the respondents were with stress, 33.3% with anxiety, and 46.92% with depression (Khan et al., 2020). The emerging mental health issues are often accompanied by abnormal behaviors. For instance, a study on Turkish university students showed that 90% of 3,040 respondents reported an increase in handwashing due to the outbreak, and 50% respondents reported that they wanted to wear protective gloves for everything they did (Akdeniz et al., 2020). At present, the mental health problems of college students during the COVID-19 pandemic have attracted the attention of relevant researchers. The current research has shown that during the pandemic, the mental health of college students has been affected to some extent, and the number of students with negative emotions and psychological problems has increased (Khan et al., 2020), revealing the possible mental health impact of COVID-19 on them. Hence, we hypothesized that (Hajivalili et al., 2020) COVID-19 would have adverse effects on the mental health of college students (World Health Organization, 2020). The prevalence of these mental health issues would be affected by nations, gender, and the survey date, etc.

Mental health instruments used by the institutes in COVID-19 related research were different, and the results varied therein; meanwhile, the reported prevalence in different nations and periods varied as well. A systematic analysis of the impact of COVID-19 on mental health of college students can obtain a more validated conclusion to assist psychological health education and mental rehabilitation of students during the pandemic. This study aimed to analyze the association between the COVID-19 epidemic and the mental health of college students with systematic review and meta-analysis and to provide the synthetic prevalence of mental health problems in college students.

## Methods

### Registration

Our study protocol was registered on the International Prospective Register of Systematic Reviews^1^ with registration number CRD42020208619. The preferred reporting items for systematic reviews and meta-analyses (PRISMA) were followed (Moher et al., 2010).

### Data Sources and Search Strategies

The searches were conducted in nine electronic databases (PsyclNFO, MEDLINE, Scopus, PubMed, EMBASE, CINAHL, ERIC, CNKI, World Health Organization Collaborating Centres Database, and Portal) with the search strategies that combined the search terms: (2019 novel coronavirus-infected pneumonia or 2019 novel coronavirus or 2019 novel coronavirus pneumonia or COVID-19 pneumonia or COVID-19 or 2019-nCOV) and (undergraduate or academician or university student or college student or higher education students) and (mental health or mental disorder or mental wellbeing or psychological health or psychology distress or mental illness or mental disorder or mental health problem or emotional health or emotion regulation or cognitive reappraisal or expressive suppression or subjective wellbeing or life satisfaction or depression or anxiety), search in December 2019 and October 2020. In addition, we have searched the literature included in the references, which was subject related and included by systematic reviews so as to supplement, obtain relevant literature, and ensure the recall ratio.

### Eligibility Criteria

Eligible studies must meet the following inclusion criteria; original studies on mental health among current college students (college students in this study specifically referred to those who are in the stage of receiving higher education, without including students who have temporary absences from school, dropping out of school, etc.) in the COVID-19 pandemic; observational studies that measured the behaviors of college students in the COVID-19 pandemic; studies that assessed the mental health status of college students using validated mental health assessment tools with good reliability and validity, such as self-rating depression scale (SDS), patient health questionnaire-9 (PHQ-9) and generalized anxiety disorder (GAD-7), whose positive rate was determined by the scoring standard of each evaluation tool. We excluded review studies and randomized controlled trials, case reports, studies with methodological bias, unified data repeated publication, sampling locations not reported, and conflicting results after a full text reading. There were no restrictions regarding language.

### Literature Screening and Data Extraction

The literature was screened and then the data were extracted by two investigators, respectively (YL and AW). The results were cross-checked. Any disagreements were resolved by the consultations with the third independent reviewer (HH).

The content from data extraction included (i) basic information of the studies (e.g., first author, publication time, research nation, sample size, and mental health problems of the survey), (ii) basic characteristics of the participants in the studies (e.g., age, gender, major, and education level), (iii) tools for evaluating mental health, (iv) outcome indicators (e.g., depression, anxiety, and other psychological problems), (v) key elements of bias risk evaluation (e.g., inclusion criteria of the research sample, whether the research identifies confounding factors, whether the research controls confounding factors, and data analysis methods).

### Assessment of Risk of Bias

We assessed the qualities of the studies with Joanna Briggs Institute (JBI) checklist for each study design (Rebecca et al., 2019). The checklist consists of eight evaluation items, which were used to evaluate the literature quality and methodological quality of studies. The evaluation of each item was divided into four categories (yes, no, unclear, and not applicable), and the judgment was based on the degree of conformity of the items. The overall evaluation of the included articles was obtained by synthesizing the evaluation of eight items (include, exclude, and seek further info) (Moola et al., 2020). The evaluation of the quality of included studies was independently evaluated by two researchers. In case of disagreements, they were resolved through discussions or negotiations with a third party.

### Data Synthesis and Statistics

Stata, version 15.1 (StataCorp.), was utilized to collect data and to perform relevant analyses in this meta-analysis (Gebrie et al., 2019; Adane et al., 2020). Each numerical value of the result was presented with a 95% confidence interval (95% CI). Publication bias was measured through Egger’s and Begg’s tests; *p* < 0.05 was considered a significant publication bias. In addition, a sensitivity analysis was performed to test the result stability, using Stata 15.1 software.

Q test and I-square statistics were utilized to test the heterogeneity across the included studies. A fixed-effects model was used when the heterogeneity was no significant between studies (I-square < 40%, *P* < 0.1) for meta-analysis; otherwise, a random-effects model was used if the heterogeneity was significant (I-square ≥ 40%, *P* < 0.1) for meta-analysis (Huggings and Sally, 2008), and subgroup analysis or sensitivity analysis was performed to explore heterogeneity (Zhao et al., 2017). According to the research characteristics of the included studies and the potential factors that affect the mental health of college students, we conducted a subgroup analysis based on the survey nations (China or non-China), gender (male or female), education level (undergraduate or graduate), major (medical or non-medical), and survey date (before or after March 1).

## Results

**FIGURE 1:**
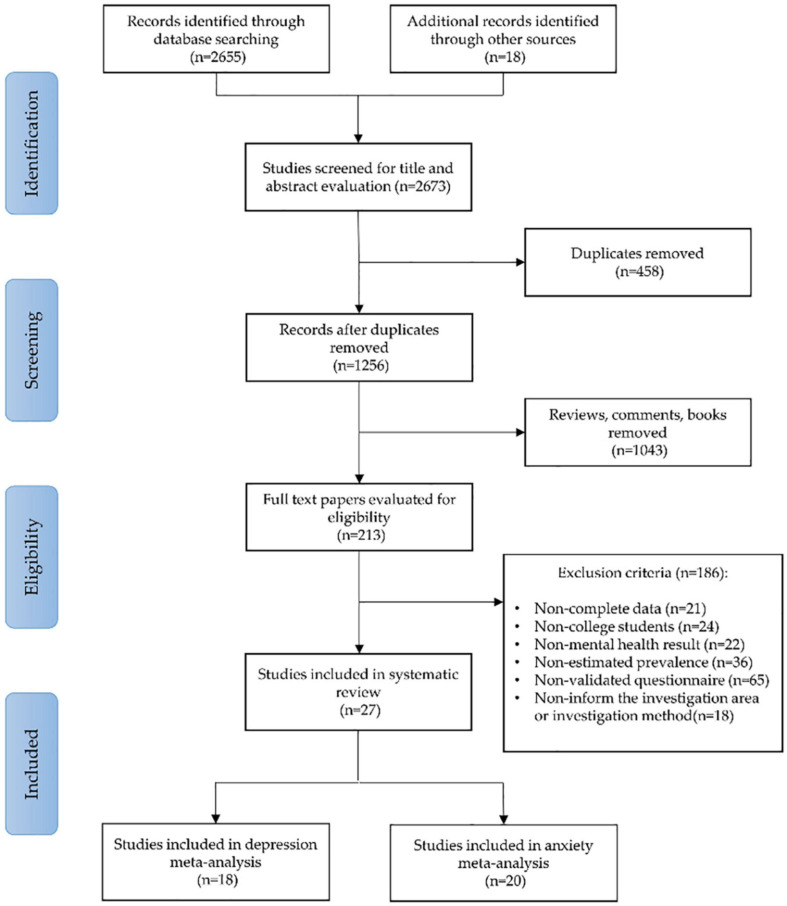
A flowchart describing the search strategy and selection of studies, using the preferred reporting items for systematic reviews and meta-analyses (PRISMA).

A total of 2,673 studies were obtained through a preliminary search. A total of 959 repeated articles were eliminated, 458 short papers were eliminated, 1,043 articles were eliminated after reading their titles and abstracts, and 186 articles were excluded after reading the full text. Eventually, a total of 27 articles were included in the meta-analysis (Huggings and Sally, 2008; Zhao et al., 2017; Bo et al., 2020; Cao et al., 2020; Chang et al., 2020; Chen R.N. et al., 2020; Dratva et al., 2020; Díaz-Jiménez et al., 2020; Fawaz and Samaha, 2020; Gilbert et al., 2020; Husky et al., 2020; Islam et al., 2020; Jia et al., 2020; Jiang et al., 2020; Mechili et al., 2020; Naser et al., 2020; Rudenstine et al., 2020; Tang et al., 2020; Wang and Zhao, 2020; Wang K. et al., 2020; Wang X. et al., 2020; Wang Z.H. et al., 2020; Wu et al., 2020; Xiao et al., 2020; Xinli et al., 2020; Yao et al., 2020; Zhan et al., 2020). The number of subjects who participated in the quantitative analysis was 706,415 college students. The search process and the selection phases are illustrated in the Flow Diagram, following the PRISMA protocol (Figure 1).

### Characteristics of the Studies and Methodological Quality

The characteristics of the 27 studies included in this review are shown in Table 1. Regarding the distribution of survey nations included in the studies, 15 studies surveyed Chinese nations, 11 studies surveyed non-Chinese nations, 1 study investigated three nations (China, Japan, and South Korea). Except for the four studies that did not report the investigation time, the earliest investigation time was January 31, 2020, and the latest investigation time was May 4, 2020. A total of 706,415 participants were included in 27 studies. The study that has the minimum number of participants has 84 participants, while the study that has the maximum number of participants has 320,000 participants. Except for a study of 477 people that did not report gender, there were 284,478 men, 421,433 women, and 27 others. Among the included studies, six studies did not report the age of the participants, and the rest of the studies had survey subjects over 17 years old. Concerning the educational level, 18 studies distinguished whether the survey subjects were undergraduates or graduate students, and 9 studies did not report it. Regarding the majors, 6 studies indicated the participants’ majors, and 21 studies did not report. The included 27 studies reported a total of 14 types of mental health issues or symptoms, of which anxiety (21 studies) and depression (19 studies) were the most reported ones, while none of the other psychological problems were reported by more than four studies.

**TABLE 1:** Characteristics of the studies and methodological quality.

Number	Author location	Survey time	Sample male/female	Age^1^	Educational level	Majors	Instrument	Main outcomes	JBI evaluation results
1	Chen R.N. et al., 2020 China	Feb. 13th–Feb. 22nd	323489 130516/192973	19−22	Undergraduate	NM	PHQ-9, RESEs	B, D, L, H	Include
2	Cao et al., 2020 China	NM	7143 2168/4975	NM	Undergraduate	NM	GAD-7	A	Include
3	Wang and Zhao, 2020 China	Feb. 10th–Feb. 17th	3611 1454/2157	18−24	Undergraduate	Arts Sciences	SAS	A, F, I, N	Include
4	Jia et al., 2020 China	Feb. 23rd–Apr. 2nd	217 90/127	18−27	Undergraduate Postgraduate	NM	PHQ-9, GAD-7	A, D	Include
5	Husky et al., 2020 French	NM	291 72/219	19.07 ± 1,7	NM	Social Sciences^2^ Health Sciences	GAD-7	A, E	Include
6	Mechili et al., 2020 United States	Mar.30th–Apr. 9th	863 98/765	NM	Graduate student	NM	PHQ-9	D	Include
7	Xiao et al., 2020 China	Feb. 4th–Feb. 12th	933 279/654	Over 17	Undergraduate Postgraduate	NM	PHQ-9, GAD-7	A, D	Include
8	Naser et al., 2020 Jordan	Mar. 22nd–Mar.28th	1165 538/627	NM	Undergraduate Postgraduate	NM	PHQ-9, GAD-7	A, D	Include
9	Xinli et al., 2020 China	Feb. 12th–Feb. 17th	2038 755/1283	20.56 ± 1.9	NM	NM	PHQ-9, PTGI, PCL Z-SAS	A, D, G, J	Include
10	Tang et al., 2020 China	Feb. 20th–Feb. 27th	2485 960/1525	19.81 ± 1.55	Undergraduate	NM	PHQ-9, PCL-C	D, G	Include
11	Wang Z.H. et al., 2020 China	Jan. 31st–Feb. 5th	44447 20217/24230	21.0 ± 2.4	Undergraduate Postgraduate	NM	PHQ-9 SAS	A, D	Include
12	Gilbert et al., 2020 United States	Apr. 24th–Jun. 5th	477 NM	20.7	NM	NM	GAD-2, PHQ-2	A, D, K	Include
13	Zhao et al. Bo et al., 2020 South Korea, China, Japan	Mar. 23rd–Apr.20th	821 305/516	23.08 ± 4.78	Undergraduate Postgraduate	NM	PHQ-9	A	Include
14	Wang X. et al., 2020 United States	May. 4th–May.19th	2031 779/1252	22.88 ± 5.22	Undergraduate Postgraduate	NM	PHQ-9 GAD-7	A, D, H	Include
15	Díaz-Jiménez et al., 2020 Spain	May. 1st – May. 24th	365 36/329	23.22 ± 6.16	Undergraduate Other	NM	DASS-21 (Spanish)	A	Include
16	Islam et al., 2020 Bangladesh	May. 6th–May. 12th	476 320/156	Over 17	NM	NM	PHQ-9 GAD-7	A, D	Include
17	Rudenstine et al., 2020 United States	Apr. 8th–May. 2nd	1821 493/1301	26.17	Undergraduate Postgraduate	NM	PHQ-9, GAD-7	A, D	Include
18	Fawaz and Samaha, 2020 Lebanese	Apr. 20th–Apr. 27th	520 201/319	21.03 ± 2.66	Undergraduate	NM	DASS-21	A, D	Include
19	Dratva et al., 2020 Swiss	Apr. 3rd–Apr. 14th	2429 753/1676	26.40 ± 5.40	Undergraduate Postgraduate	NM	GAD-7	A	Include
20	Chang et al., 2020 China	Jan. 31st–Feb. 3rd	3881 1434/2447	Over 18	NM	NM	PHQ-9, GAD-7	A, D	Include
21	Wang K. et al., 2020 China	Feb. 13th–Feb.16th	430 139/291	18−25	NM	Medical Specialty Non-Medical Specialty	SAS, SDS	A, D	Include
22	Jiang et al., 2020 China	Feb. 27th–Feb. 29th	399 162/237	NM	NM	Medical Specialty	PHQ-9, GAD-7	A, D	Include
23	Zhan et al., 2020 China	Mar. 17th–Mar. 19th	266 76/190	NM	Undergraduate Postgraduate	Medical Specialty	DASS (China)	A, D	Include
24	Yao et al., 2020 China	Feb.27th–Feb. 28th	84 52/32	19.9 ± 2.21	Undergraduate	Non-Medical Specialty	PHQ-9, GAD-7	A, D	Include
25	Wu et al., 2020 China	Feb. 16th–Feb. 20th	1196 402/794	Over 17	Undergraduate	NM	SAS	A, D	Include
26	Zolotov et al., 2020 Israeli	NM	370 77/289^ 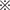 ^	25.2 ± 3.1	NM	NM	FCV-19s	F	Include
27	Xueguo et al., 2020 China	NM	304167 122102/182065	NM	NM	NM	IES-6	E, G, O, P	Include

*^1^Age was expressed as a mean ± standard deviation (M ± SD) or range.^2^Social sciences included social sciences, technology and law and economics.NM: not mentioned; PHQ-9: patient health questionnaire-9; RESEs: regulatory emotional self-efficacy scale; GAD-7: generalized anxiety disorder-7; SAS: self-rating anxiety scale; PCL: PTSD checklist; PTGI: posttraumatic Growth Inventory; Z-SAS: Zung Self-Rating Anxiety Scale; PCL-C: PTSD Check List-Civilian Version GAD-2: Generalized Anxiety Disorder 2; PHQ-2: Patient Health Questionnaire-2; SDS: self-rating depression scale; DASS: depression anxiety stress scale; DASS (Spanish): depression anxiety stress scale in Spanish; DASS (China): depression anxiety stress scale in China; FCV-19s: fear of COVID-19 scale; IES-6: impact of events scale 6; A: anxiety; B: regulatory emotional self-efficacy; C: self-perceived mental health; D: depression; E: stress; F: fear; G: PTSD; H: suicide; I: panic; J: posttraumatic growth; K: psychological distress; L: regulatory emotional self-efficacy; M: somatic symptom; N: tired; O: sleeplessness; P: self-perceived mental health.*

Based on the JBI critical appraisal checklist for analytical cross-sectional studies, all of the articles have no methodological defect and significant risk of bias and meet the requirements of inclusion (Table 1). The “Methods of Exposure Factor Prediction,” “Measurement Methods of Outcome Indexes,” and “Diagnosis of Diseases” of the 27 included articles all adopted valid, reliable, and objectively consistent methods. Fifteen articles clearly defined the sample inclusion criteria, 19 articles identified and controlled confounding factors, and 17 articles had appropriate and sufficient analysis methods. However, among the included articles, only five articles clearly described the research participants, and the remaining 22 had unreported content in the description of the research participants.

### Meta-Analysis of Depression Prevalence

**FIGURE 2:**
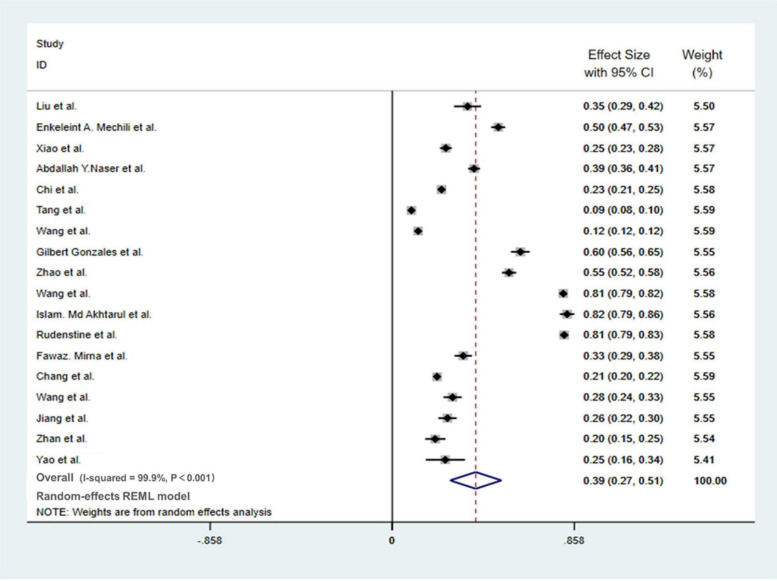
Meta-analysis of depression in college students during the COVID-19 pandemic.

Among the 19 studies on the depression of college students during the pandemic, one (Chen R.N. et al., 2020) study only reported the overall trend of the depression of participants and did not report the exact occurrence of symptoms, so this report was not included in the meta-analysis. A total of 18 studies were integrated into the meta-analysis, covering a total of 63,317 respondents. Depending on the meta-analysis (Figure 2), the prevalence of depression among college students during the pandemic was 39% (95% CI: 27–51%). The heterogeneity among the 18 studies was relatively large (I-SQUARE = 99.9%, *P* < 0.01). According to the result of the random-effects model, we found that the reasons for the heterogeneity may be complicated, and we believe that further research is indispensable.

No evidence of publication bias among the studies was observed using Begg’s test and Egger’s test (Begg’s, Pr > |z| = 0.705; Egger’s, P > | t| = 0.47; 95% CI −0.3 to.14).

### Subgroup Analysis

Subgroup analysis was operated in terms of the survey nation, gender, educational level, major, and survey date. Table 2 shows that non-Chinese college students (60%, 95% CI: 46–74%) have higher prevalence of depression compared with Chinese college students (26%, 95% CI: 21–30%), and the surveys conducted after March 1 (54%, 95% CI: 40–67%) have higher prevalence of depression compared with the surveys operated before March 1 (21%, 95% CI: 16–25%). In our research, we found that the prevalence of depression was closed for men and women, for undergraduate and graduate students, and for medical and non-medical majors.

**TABLE 2:** Subgroup analysis of depression in college students during the COVID-19 pandemic.

Subgroup	Researches (Num)	Heterogeneity		Model	Meta-analysis
		I-square	*P*		Effect Size (95% CI)
**Survey nation**					
China nations	10	98.8%	<0.05	REM	0.26 (0.21–0.30)
Non-China nations	8	99.5%	<0.05	REM	0.60 (0.46–0.74)
**Gender**					
Male	5	72.6%	<0.05	REM	0.22 (0.17–0.28)
Female	5	84.3%	<0.05	REM	0.25 (0.19–0.32)
**Educational level**					
Undergraduate	7	99.1%	<0.05	REM	0.23 (0.16–0.30)
Postgraduate	5	96.4%	<0.05	REM	0.22 (0.14–0.31)
**Major**					
Medicine	5	87.6%	<0.05	REM	0.24 (0.19–0.30)
Non- Medicine	2	78.4%	<0.05	REM	0.18 (0.06–0.31)
**Survey Date**					
By Mar. 1st	8	98.7%	<0.05	REM	0.21 (0.16–0.25)
After Mar. 1st	10	99.5%	<0.05	REM	0.54 (0.40–0.67)

*REM, random-effects model.*

### Meta-Analysis of Anxiety Prevalence

**FIGURE 3:**
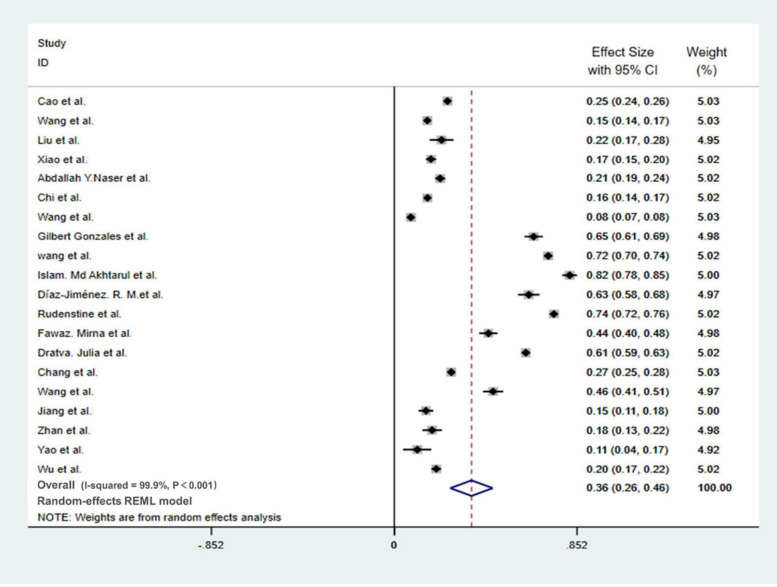
Meta-analysis of anxiety in college students during the COVID-19 pandemic.

Among the 21 studies on the depression of college students during the pandemic, one (Husky et al., 2020) study only reported the overall trend of anxiety of the respondents while did not report the specific occurrence of symptoms, so this report was not included in the meta-analysis. A total of 20 studies were included in the meta-analysis, with 73,912 participants. The meta-analysis showed (Figure 3) that the prevalence of depression among college students during the pandemic was 36% [95% CI (26%, 46%)]; the results showed that the heterogeneity among the 20 studies was relatively large (I-SQUARE = 99.9%, *P* < 0.01), According to the result of the random-effects model, we found that the reasons for the heterogeneity may be complex; we believe that further investigation is needed.

No evidence of publication bias among the studies was observed using Begg’s tests and Egger’s tests (Begg’s, Pr > | z| = 0.871; Egger’s, P > | t| = 0.308; 95% CI: −0.36 to.12).

### Subgroup Analysis

Subgroup analysis was operated in terms of the survey nation, gender, educational level, major, and survey time. During the outbreak period, the prevalence of anxiety shown by non-Chinese college students (60%, 95% CI: 46–74%) was significantly higher compared with that of Chinese college students (20%, 95% CI: 14–26%), and the prevalence of anxiety of the subgroups who completed the survey after March 1 (37%, 95% CI: 26–48%) was higher than that of the subgroups who completed the survey before March 1 (19%, 95% CI: 13–25%). However, there are no significant differences among the gender subgroups, educational level subgroups, and major subgroups (Table 3).

**TABLE 3:** Subgroup analysis of anxiety in college students during the COVID-19 pandemic.

Subgroup	Researches (Num)	Heterogeneity		Model	Meta-analysis
		I-square	*P*		effect size (95% CI)
**Survey nation**					
China nations	12	99.5%	<0.05	REM	0.20 (0.14–0.26)
Non-China nations	8	99.6%	<0.05	REM	0.60 (0.46–0.74)
**Gender**					
Male	7	99.2%	<0.05	REM	0.27 (0.12–0.42)
Female	8	99.1%	<0.05	REM	0.33 (0.22–0.43)
**Educational level**					
Undergraduate	10	99.6%	<0.05	REM	0.23 (0.16–0.30)
Postgraduate	5	96.3%	<0.05	REM	0.17 (0.09–0.25)
**Major**					
Medicine	5	18.9%	0.294	REM	0.17 (0.15–0.19)
Non- Medicine	3	46.9%	0.152	REM	0.16 (0.12–0.20)
**Survey time**					
By Mar. 1st	9	99.4%	<0.05	REM	0.19 (0.13–0.25)
After Mar. 1st	8	99.9	<0.05	REM	0.37 (0.26–0.48)

*REM, random-effects model.*

### Sensitivity Analysis

For the purpose of verifying the robustness of the meta-analysis, we conducted a sensitivity analysis by excluding the articles one by one in each step and then obtained the meta-analysis results of the remaining studies. During sensitivity analysis, the included studies were excluded one by one; we got almost the same results, which show that the studies on depression and anxiety both had good stability (Table 4).

**TABLE 4:** Sensitivity analysis.

Excluding	Effect size	95% CI
**Depression**		
Liu et al.	0.39	0.27−0.51
Enkeleint A. Mechili et al.	0.38	0.26−0.50
Xiao et al.	0.40	0.28−0.52
Abdallah Y. Naser et al.	0.39	0.26−0.52
Chi et al.	0.40	0.27−0.53
Tang et al.	0.41	0.27−0.55
Wang et al.	0.40	0.26−0.54
Gilbert Gonzales et al.	0.38	0.26−0.50
Zhao et al.	0.38	0.26−0.50
Wang et al.	0.37	0.27−0.47
Islam. MdAkhtarul et a	0.36	0.24−0.48
Rudenstine et al	0.36	0.26−0.36
Fawaz. Mirna et al.	0.40	0.26−0.54
Chang et al.	0.40	0.27−0.53
Wang et al.	0.40	0.27−0.53
Jiang et al.	0.40	0.27−0.53
Zhan et al.	0.40	0.27−0.53
Yao et al.	0.40	0.28−0.52
Overall	0.39	0.27−0.51
**Anxiety**		
Cao et al.	0.36	0.25−0.47
Wang et al.	0.37	0.26−0.48
Liu et al.	0.36	0.26−0.46
Xiao et al.	0.37	0.27−0.47
Abdallah Y. Naser et al.	0.37	0.27−0.47
Chi et al.	0.37	0.26−0.48
Wang et al.	0.37	0.27−0.47
Gilbert Gonzales et al.	0.34	0.24−0.44
wang et al.	0.34	0.25−0.43
Islam. MdAkhtarul	0.33	0.24−0.42
Díaz-Jiménez. R. M.	0.34	0.24−0.44
Rudenstine et a	0.34	0.25−0.43
Fawaz. Mirna et al	0.36	0.25−0.47
Dratva. Julia et al	0.35	0.25−0.45
Chang et al.	0.36	0.25−0.47
Wang et al.	0.36	0.26−0.46
Jiang et al.	0.35	0.25−0.45
Zhan et al.	0.37	0.27−0.47
Yao et al.	0.37	0.27−0.47
Wu et al	0.37	0.27−0.47
Overall	0.36	0.26−0.46

### Publication Bias

**FIGURE 4:**
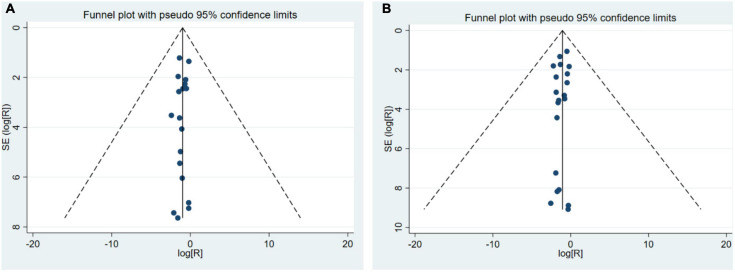
A funnel plot **(A)** A funnel plot of depression prevalence; **(B)** A funnel plot of anxiety prevalence.

The funnel plot was applied to evaluate the publication bias of depression and anxiety research, respectively. Funnel plots assessing the risk of publication bias showed symmetric distribution, indicating a lack of publication bias (Figure 4).

### Other Mental Health Issues or Symptoms

In addition to depression and anxiety, another 12 types of psychological issues or symptoms (including stress, posttraumatic stress disorder, suicidal tendencies, fear, panic, posttraumatic growth, psychological distress, regulatory emotional self-efficacy, somatic symptoms, tiredness, sleeplessness, and self-perceived mental health) were reported (Table 5). Concerning the investigations of the stress of college students during the pandemic, 3 studies were involved, and a total of 305,244 participants were surveyed, of which 56,239 participants showed positive results. In the 3 studies on college students, stress disorder during the period of the outbreak (308,690 students surveyed); there were 99,961 students showing positive and 23,299 interviewed participants reporting that they had or currently have “suicidal” thoughts (2 studies, 325,520 students were interviewed).

**TABLE 5:** A list of investigation of psychological problems.

Psychological problems	Researches	Participants	Patients	Analytical method	Effect size
Depression	19	386,806	37,714	^1^Meta-analysis	39% (95% CI: 27–51%)
Anxiety	21	74,203	13,746	^2^Meta-analysis	36% (95% CI: 26–46%)
Stress	4	305,244	56,239	NMA	6.39–21.65%
PTSD	3	308,690	99,961	NMA	2.70–32.74%
Suicide	2	325,520	23,299	NMA	7.09–18.02%
Fear	2	3,981	452	NMA	12.52%
Panic	1	3,611	737	NMA	20.41%
PTG	1	2,038	1,363	NMA	66.88%
PD	1	477	290	NMA	60.80%
RESE	1	323,489	NM	NMA	^3^None
Somatic Symptom	1	84	9	NMA	10.71%
Tired	1	3,611	731	NMA	20.24%
Sleeplessness	1	304,167	9,752	NMA	3.21%
Self-perceived Mental Health	1	304,167	1,565	NMA	0.51%

*^1^Meta-analysis was shown in Figure 2.^2^Meta-analysis was shown in Figure 3.^3^There was no effective effector.NM, not mentioned; NMA, no merge analysis; PTSD, posttraumatic stress disorder; PTG, posttraumatic growth; PD, psychological distress; RESE, regulatory emotional self-efficacy.*

## Discussion

The public was not psychologically prepared for the pandemic due to the nature of suddenness, severity, and negativity of the emergency. In order to control the spread of the pandemic, many restricted local prevention policies (restricted going out, restricted visiting relatives and friends, restricted gatherings, etc.) had been taken although they affected the normal lives of the people (Barnali and Tathagata, 2020; Quadros et al., 2020; Yanmengqian et al., 2020). With a steadily increasing number of confirmed cases and deaths of COVID-19, the infections of relatives around them, the spread of rumors, and the long-term social isolation have caused the public to experience different degrees of physical and psychological problems. This study found that depression and anxiety were the most reported psychological problems in the research on the impact of new coronary pneumonia on the mental health of college students. Meanwhile, excessive stress, posttraumatic stress disorder, psychological panic, posttraumatic growth disorder, psychological distress, emotional self-management disorder, suicidal tendency, insomnia, somatization, fatigue, and inadequate mental health status were considered to be potential mental health risks for college students.

This study showed that during the COVID-19 pandemic, the prevalence of depression among college students was 39% [95% CI (27%, 51%)]. According to WHO statistics, the global prevalence of depression in 2015 was 4.4% (World Health Organization, 2017). In addition, Global Burden of Disease Study 2017 (GBD-2017) (Kyu et al., 2018) revealed that there are significant differences in the prevalence of depression in different nations and different ethnic characteristics, but the prevalence of depression in most countries is less than 35%. College students are a special group that should not be ignored during the COVID-19 pandemic, whose mental health is affected by many factors; therefore, the prevalence of depressive symptoms in college students is not only higher than that in other groups but also has individual differences (Happell et al., 2020). The prevalence of depression in college students during the pandemic was analyzed by the regional subgroup, the gender subgroup, the educational level subgroup, the major subgroup, and the survey date. The results showed that the prevalence of depression in college students during the pandemic was similar among the gender subgroup, the educational level subgroup, and the major subgroups, but the difference was found in the subgroup analysis of the survey areas and the survey date; that is, the prevalence of depression in non-Chinese areas (60%) was greater than in China (26%), and the rate of depression completed after March 1 (54%) was greater than that of those completed before March 1 (21%). A meta-analysis of 39 studies from 1997 to 2015 showed that the pooled prevalence of depression in Chinese college students for depression reached 23.8% (Lei et al., 2016), which was lower than that reported in our study (26%). In the study of the occurrence of depressive symptoms in college students in non-Chinese nations, studies have shown that the prevalence of depressive symptoms in college students in non-Chinese nations is 49.7%, while the prevalence of depressive disorders is 16.7% (Newhart et al., 2019). In 2016, a mental health survey of 67,308 undergraduate students in the United States conducted by the American University Health Association showed that 13.4% of the students were depressed (Liu et al., 2019), and, meanwhile, the prevalence of depression in non-Chinese college students in this study (60%) was higher than 49.7 or 13.4%. Although there are differences in the prevalence of depression among students in different nations, the results showed that the prevalence of depression among college students has increased during the pandemic.

Concerning anxiety, the results of this study show that the prevalence of it among college students during the pandemic was 36% [95% CI (26%, 46%)], which was higher than the global prevalence of anxiety (3.6%) in 2015 according to WHO statistics (World Health Organization, 2017). Anxiety and depression have a high comorbidity rate and have some common symptoms, including fatigue, irritability, difficulty concentrating, and sleep disorders, suggesting that the two have some common psychopathological bases (Kalmbach et al., 2017; Tang et al., 2018). A survey involving 7,402,045 people across 24 countries found that 45.7% of depression patients are also suffering from anxiety disorder (Kessler et al., 2015); 60–70% of patients with generalized anxiety disorder have had an episode of depression in their lifetime (Adams et al., 2016). As a result, depression and anxiety not only bring more pain to the patient but also make the treatment more difficult. Similarly (Rebar et al., 2017), the degree of anxiety differs greatly based on nations and individuals (Suran et al., 2016). The subgroup analysis of this study shows that the prevalence of anxiety among college students during the pandemic has relatively smaller differences in gender subgroups, educational level subgroups, and major subgroups, while it has large differences in regional subgroups and survey date subgroups. The prevalence of anxiety among college students in non-China regions was (60%) higher than that of students in China (20%), and the prevalence of anxiety after March 1 (37%) was higher than that of students surveyed before March 1 (19%). Anxiety prevalence was higher in different regions and at different survey dates during the pandemic than during normal periods (Tang et al., 2018; Aljawawdeh and Alghazo, 2019; Melissa et al., 2019; Saba et al., 2020).

In public health emergencies, the public psychological reactions related to the epidemic situation are often influenced by region and temporal distributions. A recent study has shown that the prevalence of mental disorders in Chinese public was relatively low in the early stages of the COVID-19 pandemic. In addition, the mental state of people was affected by geographical and temporal distributions (Ren et al., 2020). The results of this study showed that there were obvious regional differences and survey date differences in the mental health status of college students during the pandemic. In China, except for the staff fighting against the pandemic, almost everyone experienced self-isolation, which meant that people had to stay at home due to strict lockdown and restriction policy. Strict quarantine policy allowed the pandemic in China to be well controlled, which increased the confidence of Chinese people in defeating the new crown pneumonia pandemic, thereby reducing the occurrence of psychological problems (Hien et al., 2020; Pan et al., 2020). Countries outside of China were relatively late in the outbreak of the pandemic, and insufficient attention was paid to the prevention of the pandemic at the beginning of the period. This increased the psychological pressure of local students, which would then cause a series of mental health problems. In addition, there are differences in the applicable populations of the mental health survey tools used in the research, or regional differences in the prevalence standards of mental health problems, which will increase the regional differences in mental health problems. In addition, as the epidemic spreads around the world, all parts of the world are caught in panic, which has increased the psychological burden of college students and increased the prevalence of their psychological problems. Studies have shown that, when people face disasters, different experience times can have different psychological effects; and long-term “disaster” environments increase the risk of psychological problems with the epidemic, and the prevalence of psychological problems among college students has gradually increased.

This study showed that compared with the male students, the prevalence of depression and anxiety in the female students during the epidemic was higher, which may be caused by the different physiological structures and functions between the male and female students. Compared with the male students, the female students were less courageous, more dependent on others, and have stronger stress responses when confronted with emergencies (Patel et al., 2007; Hyde et al., 2008; Rosenfield and Mouzon, 2013; Haugen et al., 2014). Subgroup analysis showed that medical students were more fragile than non-medical students to suffer anxiety and depression during the pandemic, which may be associated with their special major. There was a study reporting that the level of mental health status such as depression and anxiety among the university healthcare workers was steadily prevalent even after the lockdown period was lifted (Woon et al., 2020). Compared with workers in other professions, medical workers endured higher levels of burnout (Prosser et al., 1996; Tang et al., 2019). Burnout is significantly related to depression and anxiety and has a negative impact on the health of healthcare workers (Foster et al., 2019; Payne et al., 2020). However, few existing psychological studies have evaluated the mental health status of college students of different majors. Therefore, the results of this subgroup analysis need to be confirmed by further studies.

In the face of danger and strong stressor stimulation, psychological health of an individual is threatened and accompanied by panic behaviors. College students lack the ability and experience to self-regulate and self-rescue. It is inevitable that they are all under stress, which causes emotional pain and psychological fluctuations in some college students. Different students have different mental states and behavioral responses (depression, irritability, anxiety, insomnia, disappointment, and doubts, and some of them even show excessive worrying about health, repeated disinfection, repeated handwashing, drug abuse, etc.), and the state of being isolated at home makes students feel distrustful of their surroundings so that they have different levels of psychological problems such as interpersonal sensitivity, hostility, and paranoia (Goebel and Mills, 2000; Fergusson et al., 2014; Kouichi et al., 2019). College students also have to face many problems with their studies, graduations, and employment, and are a high-risk group of psychological problems (Andrews et al., 2018). During the COVID-19 epidemic, there were some changes in the mental status of college students, including the occurrence of some psychological problems (Lei et al., 2020). This study summarizes the negative changes in the mental state of college students during COVID-19 and indicated the mental health problems and potential mental health risks college students had the most during the COVID-19 period. This synergy was conducive to the spiritual comfort and mental health maintenance of college students during the pandemic prevention period and provided directions for psychological counseling for students after the social isolation or local control.

The COVID-19 pneumonia is highly contagious (Andrews et al., 2018), and its transmission route is respiratory-based. The homology with severe acute respiratory syndrome (SARS) virus is more than 85%, and the characteristics of the disease are unknown. It causes great psychological stresses to the population (Jia et al., 2020; Zhang, 2020). Therefore, during the special major public health crisis of COVID-19, in addition to COVID-19 preventions, more attention should be paid to the mental health of college students. Based on the results of this research, schools should pay attention to the psychological conditions of college students during and after the COVID-19 pandemic situation, should pay attention to improving the psychological qualities of college students, should alleviate the helplessness of students, and should promptly target college students with psychological disorders. In addition, some online psychotherapy methods such as telemedicine and self-help mindfulness therapy may be considered to alleviate the mental health problems of college students during the COVID-19 pandemic. College students may gain benefits from online telemedicine to improve wellness and boost coping strategy such as empathic listening, psychoeducation, or supportive therapy (Hatta, 2020). Some studies have shown that self-help mindfulness therapy can improve psychological distress, positive mental health, and academic distress in college students, as well as what was expected to increase resilience and reduce depression anxiety and stress in other adults who need psychotherapy (Javedani et al., 2017; Levin et al., 2020). In addition, the society, colleges and universities, families, and students should take effective actions in time to prevent the occurrence of adverse psychology of college students during the COVID-19 pandemic. The actions include (1) complete mental health service, such as the television media publicity of mental health care or online telemedicine service; (2) timely and accurate disclosure of epidemic information to avoid public panic; (3) remote management of students to master the health dynamics and mental health status; and (4) increase of the communication with the family to encourage one another.

In order to create a mentally healthy and friendly environment for these college students, university departments and local governments should provide some professional counseling and supportive projects (Bao et al., 2020). In addition, due to the limitations of the number and quality of included studies, the above conclusions need to be verified by more high-quality studies. More research should be conducted to determine the symptoms of depression in other countries during the COVID-19 pandemic in the coming months to provide more general data. The results of this study can provide basic information for the development of mental health plans for college students and other groups.

## Limitations

One of the limitations of this study is the differences in the assessment tools and the differences in the evaluation criterion the researchers selected according to the study location. Second, most studies were observational studies, and the patients were not randomly chosen. In addition, our ability to assess the qualities of study was limited by the fact that many studies failed to offer detailed information of selected subjects or valid data on important factors. Therefore, selection biases and confounding seem to be inevitable. Third, the heterogeneity of this study was obvious, and the great statistical heterogeneity is common in the single-rate meta-analysis because there are too many confounding factors that affect the results of the study, which needs to be analyzed further. Finally, because the sample size of included literature is not large enough, the inability to perform meta-regression is another limitation of the study.

## Conclusion

During the COVID-19 pandemic, college students had a variety of psychological problems, of which depression and anxiety were the main psychological problems. The prevalence of depression and anxiety reached 39% and 36%, respectively. The mental health problems in college students were influenced by nations and survey date. The prevalence of depression and anxiety in non-Chinese college students were 60% and 60%; in contrast, 26% and 20% in Chinese college students. The survey conducted after March 1st reported that depression and anxiety were 54% and 37% while survey before March 1st showed that they were 21% and 19%. In order to create a psychologically healthy environment for these college students, university departments, and local, and central governments should provide some professional depression counseling and supportive programs.

## Data Availability Statement

The original contributions presented in the study are included in the article/Supplementary Material, further inquiries can be directed to the corresponding author/s.

## Author Contributions

YL and HH: study concept and design. YL, YW, and NH: data acquisition, analysis, and interpretation. YL, HH, and AW: preparation of manuscript and figures. All authors contributed to the article and approved the submitted version.

## Conflict of Interest

The authors declare that the research was conducted in the absence of any commercial or financial relationships that could be construed as a potential conflict of interest.

## Footnotes

1 https://www.crd.york.ac.uk/PROSPERO/

## Supplementary Material

Supplementary Figure 1 Based on the JBI checklist quality evaluation chart.
