# Supplementary figures and images for: Impact of the COVID-19 Pandemic on the Mental Health of College Students: A Systematic Review and Meta-Analysis
Source: Front Psychol. 2021 Jul 14;12:669119. doi: 10.3389/fpsyg.2021.669119 (PMC8316976; doi:10.3389/fpsyg.2021.669119)

## SUPPLEMENTARY FIGURE 1

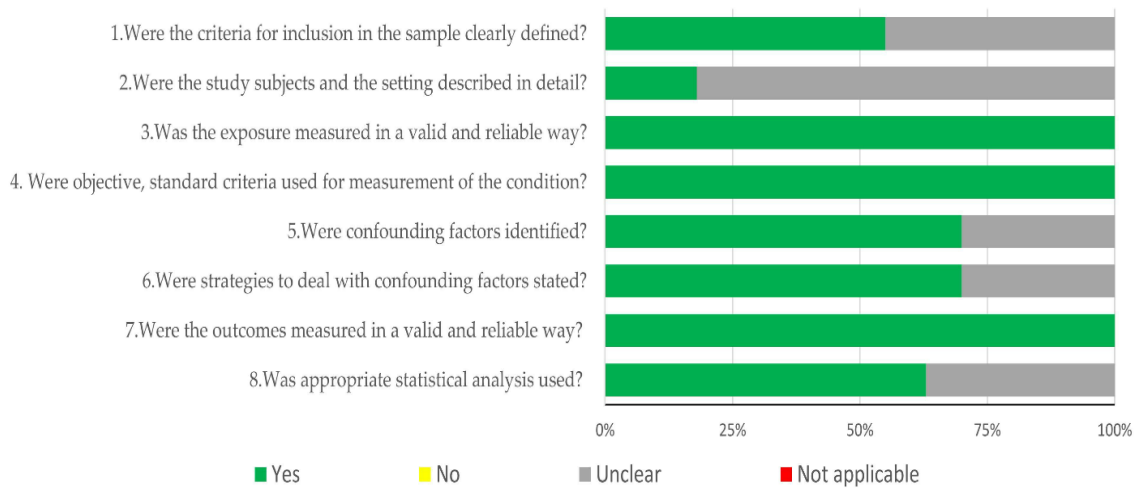

**FIGURE** Based on JBI Checklist quality evaluation chart.

Supplement: Supplementary Figure 1 — Based on the JBI checklist quality evaluation chart. [file Image_1.pdf]
